# The burden of polycystic ovary syndrome-related infertility in 204 countries and territories, 1990-2021: an analysis of the global burden of disease study 2021

**DOI:** 10.3389/fendo.2025.1559246

**Published:** 2025-06-06

**Authors:** Lan Zheng, Xiao Xu, Jin-Zhuo Zhou, Lin Hong, Yu-Feng He, Ya-Xing Fang, Bing-Bing Wang, Hui Chen, Kang-Jia Chen, Su-Su Yang, Xiao-Long Yang, Hai-Feng Pan, Shu-Guang Zhou

**Affiliations:** ^1^ Department of Gynecology, Maternal and Child Health Center of Anhui Medical University, The Fifth Affiliated Clinical College of Anhui Medical University, Anhui Women and Children’s Medical Center, Hefei, Anhui, China; ^2^ Department of Epidemiology and Biostatistics, School of Public Health, Anhui Medical University, Inflammation and Immune Mediated Diseases Laboratory of Anhui Province, Hefei, Anhui, China; ^3^ Department of Gynecology, Linquan Maternity and Child Healthcare Hospital, Fuyang, Anhui, China

**Keywords:** infertility attributed to PCOS, the global burden of disease study, the bayesian age-period-cohort model, prevalence, years lived with disability rates

## Abstract

**Objectives:**

The evolving patterns of the infertility attributed to polycystic ovary syndrome (PCOS) disease burden are being evaluated, stratified by age, region, subtype, and health condition.

**Material and methods:**

This cross-sectional investigation encompassed data from 21 regions and 204 countries from 1990 to 2021, sourced through the Institute for Health Metrics and Evaluation. The prevalence and YLDs due to infertility, overall and stratified by age, subtype, region, country, and health status. The percent change in age-standardized rates (ASR) was calculated to temporal quantify the trend of infertility burden. The Bayesian age-period-cohort (BAPC) model was employed to forecast the trends in the ASR of prevalence and YLD from 2022 to 2042.

**Results:**

Globally, the age-standardized prevalence and YLDs in infertility attributed to PCOS showed a significant increase trend from 1990 to 2021, with ASR of 34.2% (95%CI: 28.2 to 41.5%) and 32.5% (95%CI: 26.6 to 39.4%), respectively. The spatiotemporal trends in infertility attributed to PCOS varied substantially between subtypes and age groups. The burden of primary infertility peaked among individuals aged 20 to 24 years, whereas secondary infertility consistently peaked among individuals aged 35 to 44 years. The BAPC model showed that the burden of infertility will increase over the next 2 decades.

**Conclusions:**

Infertility attributed to PCOS remains to be a significant public health issue globally, with this burden varying considerably across age groups, and subtypes. Decision-makers providers must take a proactive stance in monitoring developing trends and adapting infertility screening and management protocols to different age brackets and subtypes.

## Introduction

Infertility is described as the incapacity to set up a medical being pregnant after one year or longer of consistent unprotected sexual activity (1). It affects mental and psychological well-being, leading to severe physical suffering, humiliation, and financial hardship, while also negatively impacting overall quality of life (2, 3). The World Health Organization’s most recent study on the prevalence of infertility was published in April 2023. It estimates that the global prevalence of infertility is approximately 17.5%, suggesting that one in six adult individuals worldwide suffers from infertility (4). As time goes by, a reversed demographic pyramid characterized by a rising percentage of elderly individuals and a decline in the working-age populace may stem from low fertility rates (5). It is reasonable to assume that such shifts could elevate the pressures on healthcare and social services, alter labor and consumer markets, and modify patterns of resource utilization. Furthermore, the possible economic implications, along with the formulation of efficient health, environmental, and economic strategies, can be derived from the necessary precise estimations and future predictions regarding fertility rates (6). Consequently, it is essential to explore the changes in infertility and the corresponding disease burden for the prevention and treatment of infertility.

Polycystic ovary syndrome (PCOS) represents the most frequently encountered endocrine disorder in women of reproductive age (7). The prevalence rates of PCOS fluctuate between 6% and 20%, influenced by varying diagnostic standards (8, 9). Additionally, both the prevalence and incidence of PCOS continue to rise consistently (10). One of the most common issues and physical impairments caused by PCOS for women is infertility (11). Up to 80% of women with anovulation suffer from PCOS, resulting in the most prevalent cause of ovulatory dysfunction and anovulation (12). While the specific mechanism between PCOS and infertility is yet unclear, more and more evidences point to the existence of a relationship (13). Therefore, there is a need to increase awareness of the infertility burden of PCOS and provide primary prevention and treatment of female infertility. Understanding the burden of infertility caused by PCOS can guide experts and governments in developing future plans for women’s health. This may also highlight the need for enhanced PCOS management and screening in high-burden countries.

The Global Burden of Disease Study (GBD) constitutes a comprehensive international statistical evaluation of diseases, injuries, and risk factors at global, regional, and national levels (14). The GBD 2021 database, encompassing 204 countries and regions as well as 811 subnational locations, and assessing the health burden attributable to 371 diseases and injuries and 88 risk factors, served as the foundation for the latest analysis regarding the global impact of infertility related to PCOS. Xingyu Liu et al. analyzed the worldwide burden of infertility due to PCOS, alongside its relationships with various factors, including different sociodemographic index (SDI) categories and types of infertility, using the GBD Study 2019 data from 1990 to 2019 (15).This research reviews trends in the prevalence of infertility attributed to PCOS and YLDs across various SDI regions, and it explores the reasons contributing to the substantially higher burden observed in the high SDI regions (15). However, to date, there has not yet been any investigation focused on modeling and assessing the future burden of infertility caused by PCOS. Updated information on the impact of infertility associated with PCOS is needed to support advocacy efforts and public health programs. Moreover, it is essential to investigate the connections between age groups and infertility subtypes to enhance disease prevention and treatment approaches.

## Materials and methods

### Study design

Recently, we conducted a comprehensive reassessment of the burden of infertility due to PCOS utilizing the latest GBD 2021 data. Our analysis emphasized the prevalence and YLD, as well as the ASRs, examining their trends on global, regional, and national scales. We utilized the ‘maps’ and ‘maptools’ packages in R software to visualize the analytical results. In addition, we focused on the relationship between age segments and subtypes of infertility, as well as SDI across 21 regions and 204 countries and territories. We used the ‘ggplot’ package in the R software to visualize the results. Concurrently, the BAPC model was utilized to assess the burden of PCOS associated with infertility during the following 20 years, aiming to guide initiatives for prevention and treatment through the development of policies, allocation of resources, and organization of health systems.

### Data sources

The GBD 2021 database (https://vizhub.healthdata.org/gbd-results/) includes an extensive array of health and demographic information derived from census data, surveys, registries, indicators, administrative health records, and financial data related to health (16). Additionally, the SDI data for different nations was obtained from GBD 2021. In order to estimate the prevalence of infertility caused by PCOS globally, population projections for the years 2017 to 2100 were also retrieved from the GBD 2021 dataset (17). This study aims to gather primary and secondary infertility data by isolating the disease to PCOS and subsequently concentrating on infertility. Consequently, effective data collection has been achieved for females aged 15 to 49 years experiencing infertility related to PCOS. The age-standardized prevalence data served as the basis for assessing the impact of PCOS-related infertility. To examine this issue by specific age ranges, health statistics from the GBD 2021 were utilized for females in the 15 to 49 years, divided into 5-year age groups. This study followed the principles outlined in STROBE Statement (18).Since the data used in this research was publicly accessible and did not contain any personal or sensitive information, there was no necessity for ethics approval.

### Data definition

Polycystic ovarian syndrome (PCOS) is characterized by symptoms of polycystic ovaries, irregular menstruation, and increased androgen levels (19). Infertility associated with PCOS can be divided into two categories: primary infertility and secondary infertility (20). Primary infertility refers to the inability of couples to conceive and supply the beginning of a child after attractive in unprotected sexual intercourse for more than 1 year (21). On the other hand, secondary infertility is characterized by a couple’s inability to conceive after having previously had a live birth, while remaining in a stable relationship and not using contraceptives for more than 1 year (22). Our research focused on a cohort of women with PCOS who were identified as experiencing infertility through health surveys conducted in GBD 2021.

### Measures of burden

The SDI, a comprehensive indicator, reflects the overall state of social population and economic development and is associated with the health levels of each country (23). This index, which ranges from 0 to 1, is calculated by taking the average of educational years among individuals aged 15 and older, the total fertility rates within the population under 25, and the historical distribution of per capita income (24). A higher value indicates longer average educational years, a higher fertility rate, and a greater level of lagged per capita income (25). Years lived with disability(YLD) rates were assessed by multiplying the prevalence of infertility by its corresponding disability weights, quantifying the amount of health loss associated with infertility (26).YLD rates caused by PCOS-related infertility were computed through the multiplication of the prevalence of primary infertility and its corresponding disability index(0.008), as well as the prevalence of secondary infertility and its associated disability index(0.005) (25).ASR is a method used to adjust crude rates, like prevalence or YLDs, for variations in population age structure (27). By making this adjustment, standardized comparisons between various populations or time frames are facilitated, minimizing age-related biases (28). This approach is essential in epidemiological and public health research, enhancing the validity of cross-population comparisons and trend analyses where age structure disparities could distort health outcomes.

### BAPC model for forecasting (2022–2042)

The Bayesian age-period-cohort (BAPC) model, which is rooted in Bayesian statistical theory, is employed to investigate and clarify the trends of personal characteristics within the population concerning age, period, and birth cohort (29).Utilizing the typical age distribution from the GBD and the projected population data from the World Health Organization (WHO), the BAPC model predicts the prevalence of PCOS-related infertility over the next 20 years. To forecast and visualize this model, the BAPC and INLA packages were applied (30). This robust model enabled us to produce trustworthy forecasts, offering insightful perspectives on the expected evolution of the burden of infertility associated with PCOS.

### Statistical analysis

In evaluations of infertility prevalence and YLD related to PCOS, trends have been analyzed at the global, regional, and national levels through the use of counts, ASR. Additionally, the trend of disease burden was evaluated across different types and age groups. All tests, calculations, and descriptive plotting were carried out utilizing the R software (version 4.3.1, R Foundation, Vienna, Austria).

## Results

### At the global level

In 2021, 124.7 million prevalent cases of PCOS-related to infertility were reported globally, which translates to an age-standardized point prevalence of 315.8 per 100,000 women (**Table 1**). This indicates a 34.2% increase compared to 1990(**Table 1**). In 2021, the global number of YLDs due to infertility associated with PCOS was 71.6 thousand, reflecting an age-standardized rate of 1.8 YLDs for every 100,000 women, which is up by 32.5% since 1990 (**Table 1**).

**Table 1:** Prevalent cases and years lived with disability for PCOS-related Infertility in 2021, and percentage change in age-standardized rates (ASRs) per 100000, by the global burden of Disease region, from 1990 to 2021.

	Prevalence (95% UI)			YLDs(95% UI)		
	No, in 100 thousands (95% UI)	ASRs per 100000 (95% UI)	Percentage change in ASRs from 1990 to 2021	No, in thousands (95% UI)	ASRs per 100000 (95% UI)	Percentage change in ASRs from 1990 to 2021
Global	124.7 (82.2,185.6)	315.8 (208,469)	34.2 (28.2,41.5)	71.6 (28,161.7)	1.8 (0.7,4.1)	32.5 (26.6,39.4)
High income Asia Pacific	5.6 (3.6,8.1)	691 (431.3,1002.3)	6.1 (-1.3,13.4)	3.1 (1.2,6.5)	3.9 (1.5,8.2)	5.7 (-1.9,12.9)
High income North America	10.5 (7.1,15.2)	614.1 (416.8,891.3)	32.8 (17,57.5)	6.3 (2.5,13.9)	3.7 (1.5,8.2)	30.7 (14.4,54.5)
Western Europe	12.2 (8.1,17.8)	631.4 (419,913.7)	20.1 (12.2,27.8)	7.3 (2.9,16.4)	3.8 (1.5,8.6)	19.4 (10.7,28.2)
Australasia	0.8 (0.5,1.3)	540.1 (326.2,827.8)	15.1 (1.6,28.2)	0.5 (0.2,1)	3.2 (1.2,6.8)	14.6 (0.4,30.6)
Andean Latin America	1.8 (1.2,2.6)	489.3 (321.1,727.1)	68 (30.3,112.4)	1 (0.4,2.2)	2.7 (1.1,6.2)	65.2 (27.5,108.7)
Tropical Latin America	1.4 (0.8,2.1)	109.5 (68.4,171.9)	6.2 (0.6,12.4)	0.8 (0.3,1.8)	0.6 (0.2,1.5)	4.3 (-3.2,11.4)
Central Latin America	6.9 (4.4,10.2)	499 (321.9,734.8)	18.5 (10,32.9)	3.9 (1.5,8.8)	2.8 (1.1,6.4)	16.3 (6.9,31.4)
Southern Latin America	1.2 (0.7,1.8)	327.2 (207.5,499)	55.5 (41.4,70.4)	0.7 (0.3,1.6)	1.9 (0.7,4.4)	55.8 (37.3,73.3)
Caribbean	0.7 (0.4,1)	274.3 (177.2,417.4)	23.1 (16.1,29.4)	0.4 (0.1,0.9)	1.6 (0.6,3.6)	21 (12.4,30.3)
Central Europe	0.2 (0.1,0.3)	43.3 (27.5,67.8)	22.9 (9.6,38.1)	0.1 (0,0.3)	0.2 (0.1,0.6)	21.5 (6.5,39.5)
Eastern Europe	0.5 (0.3,0.8)	51.9 (32,82.7)	29.4 (22.9,35.8)	0.3 (0.1,0.7)	0.3 (0.1,0.7)	28.7 (18.9,40.5)
Central Asia	0.5 (0.3,0.7)	91.4 (57.3,143)	37.6 (27.6,48.5)	0.3 (0.1,0.6)	0.5 (0.2,1.2)	37 (21.4,52.8)
North Africa and Middle East	13.3 (8.5,20.6)	409.8 (261.6,635.8)	33.4 (25.2,41.4)	7.8 (3,17.8)	2.4 (0.9,5.5)	29.1 (18,39.4)
South Asia	22 (13.9,33.4)	218.7 (138.6,331.6)	80.5 (68.1,95.3)	12.7 (4.8,29.1)	1.3 (0.5,2.9)	75.9 (62.9,91.6)
South East Asia	19.1 (12.4,28.8)	512.1 (333,769.9)	77.4 (65.4,91.8)	11 (4.2,24.6)	3 (1.1,6.7)	75.1 (62.4,90.6)
East Asia	19.9 (12.8,30.6)	298.2 (191.9,454.1)	78.4 (66.2,93.1)	10.8 (4,25.4)	1.6 (0.6,3.8)	80.4 (66.9,97.3)
Oceania	0.2 (0.1,0.3)	315.6 (202.9,481.9)	34.4 (22.2,47.4)	0.1 (0,0.3)	1.8 (0.7,4)	33.4 (16.9,51.4)
Western sub Saharan Africa	3.4 (2.2,5.4)	140.5 (88,220.2)	44.3 (36.2,54.3)	2 (0.8,4.5)	0.8 (0.3,1.8)	44.2 (34.4,56.3)
Eastern sub Saharan Africa	2.8 (1.8,4.4)	129.4 (81.3,201.5)	28.2 (22.6,34.8)	1.6 (0.6,3.6)	0.7 (0.3,1.6)	27.2 (19,36.1)
Central sub Saharan Africa	0.9 (0.6,1.4)	134.1 (83.2,211.6)	51.6 (38,66)	0.5 (0.2,1.2)	0.8 (0.3,1.7)	51.2 (30.3,77.3)
Southern sub Saharan Africa	0.9 (0.6,1.4)	198.9 (126.6,305.4)	20.6 (12.5,30.1)	0.5 (0.2,1.2)	1.1 (0.4,2.6)	20.4 (9.6,32.5)

95% UI, 95% uncertainty intervals.

### At the regional level

In 2021, the age-standardized point prevalence of infertility related to PCOS per 100,000 females peaked in High-income Asia-Pacific [691 (95% UI: 431.3 to 1002.3)] (**Table 1**). In 2021, High income Asia Pacific [3.9 (95% UI: 1.5 to 8.2)], Western Europe [3.8 (95% UI: 1.5 to 8.6)], and High-income North America [3.7 (95% UI: 1.5 to 8.2)] had the highest age-standardized YLD rates due to infertility (**Table 1**). The largest increases in the age-standardized point prevalence, between 1990 and 2021, were observed in South Asia [80.5% (95% UI: 68.1% to 95.3%), East Asia [78.4% (95% UI: 66.2% to 93.1%); and Asia [77.4% (95% UI: 65.4% to 91.8%), with no regions decreasing during this period (**Table 1**). In addition, the greatest increases in the age-standardized YLD rates of infertility, from 1990 to 2021, were observed in East Asia [80.4% (95% UI: 66.9% to 97.3%)], South Asia [75.9% (95% UI: 62.9% to 91.6%)] and South East Asia [75.1% (95% UI: 62.4% to 90.6%)].

### At the national level

**Figure 1:**
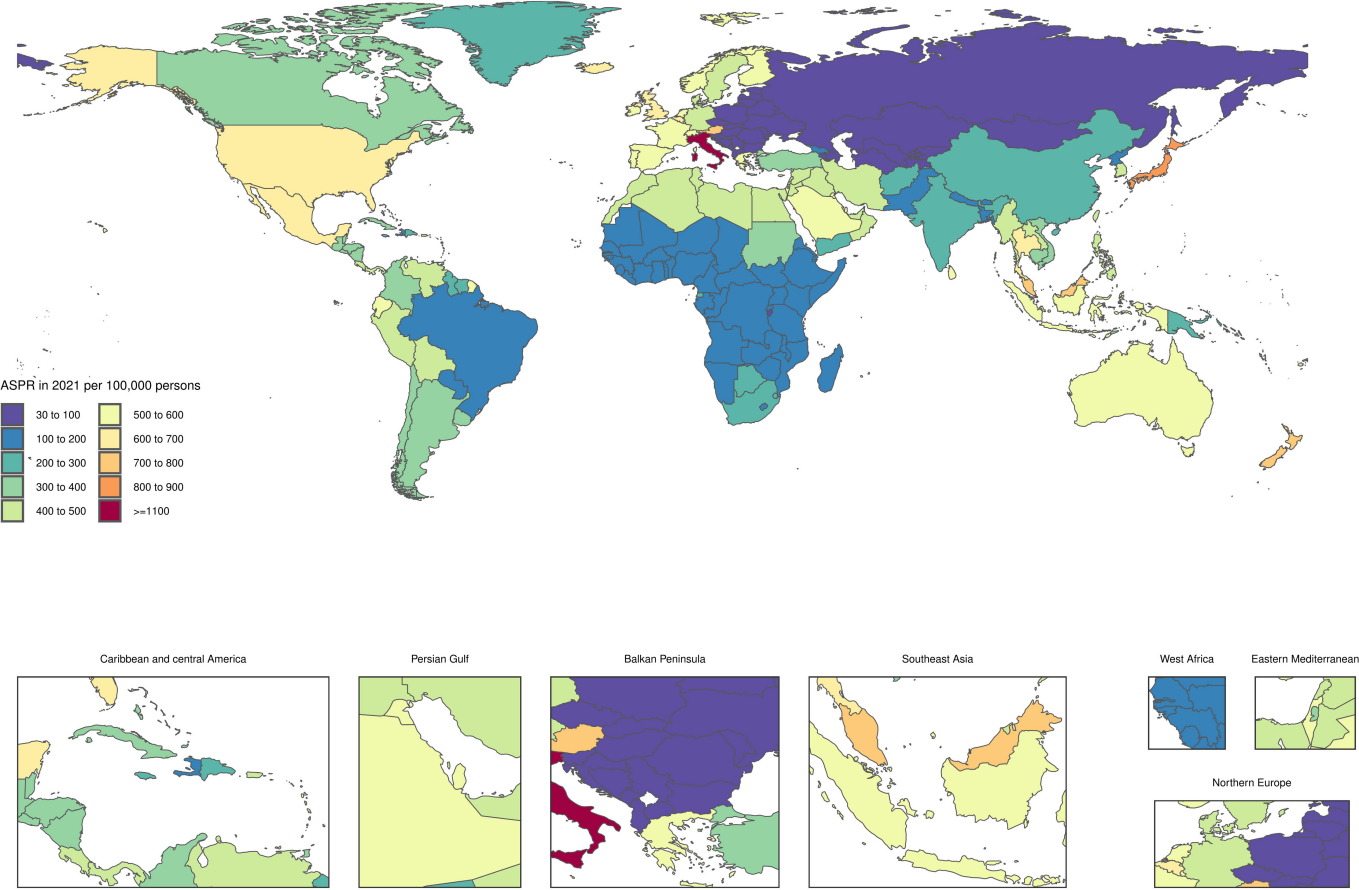
Age-standardized point prevalence of PCOS-related Infertility per 100,000 population in 2021, by countries.

**Figure 2:**
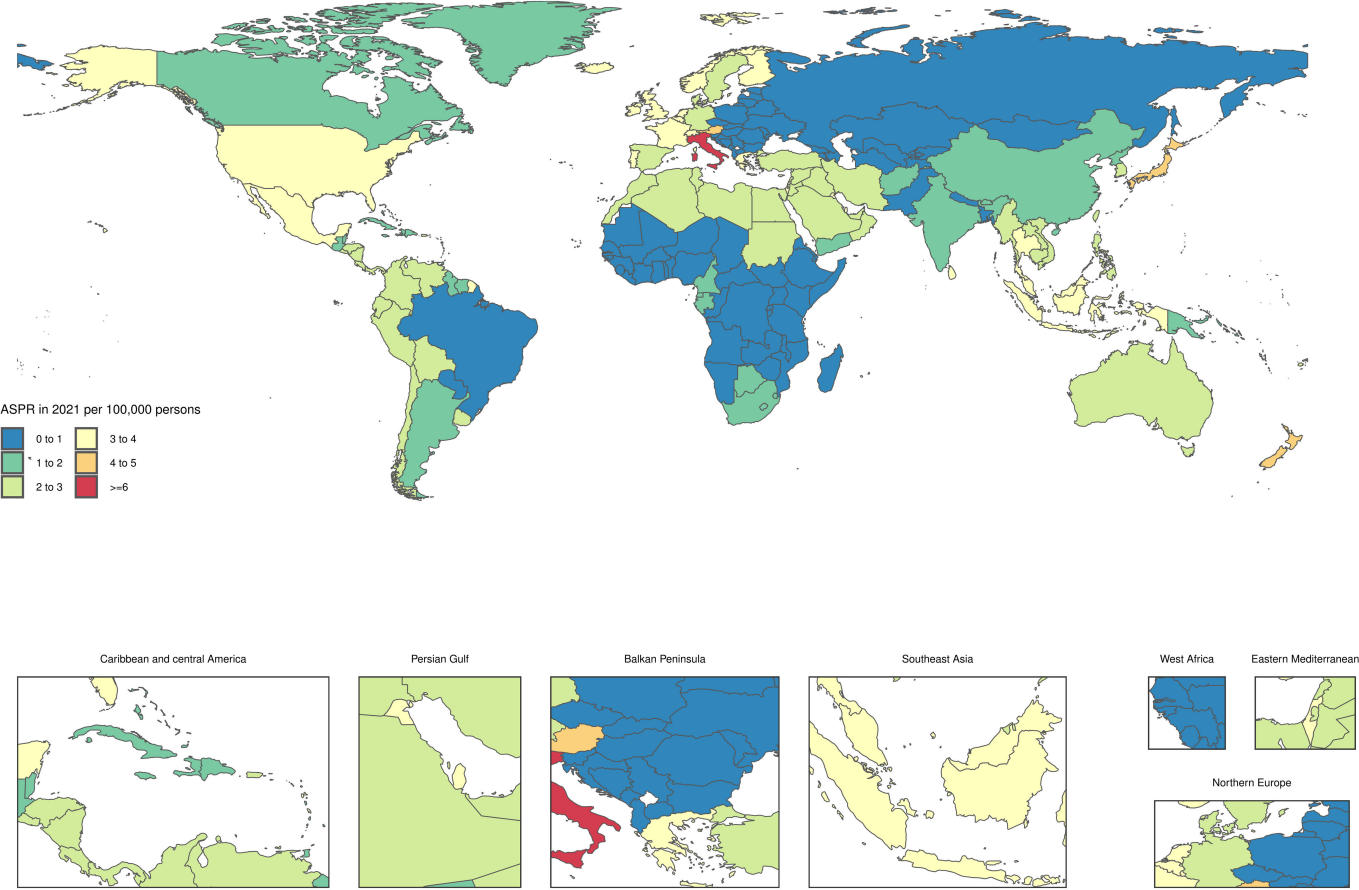
Age-standardized point YLDs of PCOS-related Infertility per 100,000 population in 2021, by countries.

In the year 2021, the age-standardized point prevalence of infertility linked to PCOS exhibited significant variation across 204 countries and territories, ranging from 33.4 to 9410 cases per 100,000 women. Notably, Japan reported the highest prevalence at 841.8 per 100,000 (95% UI: 543.3 to 1216.6), followed closely by New Zealand at 727.4 (95% UI: 461 to 1083.4) and Austria at 716.2 (95% UI: 461.2 to 1076.6) (**Figure 1**, **Supplementary Table S1**). Additionally, the age-standardized YLD rates due to PCOS were higher in certain countries. Italy led with a YLD rates of 6.9 (95% UI: 2.8 to 16), followed by Japan at 4.7 (95% UI: 1.8 to 10.2) and New Zealand at 4.3 (95% UI: 1.6 to 9.1). In contrast, several countries such as Albania, Bosnia and Herzegovina, Bulgaria, and Slovenia recorded the lowest YLD rates, with a notably low rate of 0.2 (95% UI: 0.1 to 0.5). These figures reflect the broader impacts of PCOS on women’s health and indicate variances in how the condition affects populations in different regions. Over the period from 1990 to 2021, there were also substantial changes in the age-standardized point prevalence of infertility associated with PCOS. The most significant increases were observed in Equatorial Guinea, where the prevalence surged by 132.5% (95% UI: 102.7% to 175.7%), followed by Peru with a 107.2% increase (95% UI: 59.8% to 170.8%) and the Maldives at 103.7% (95% UI: 76.8% to 133.4%) (**Figure 2**, **Supplementary Table S2**). Furthermore, the trends in the age-standardized annual YLD rates of PCOS from 1990 to 2021 revealed significant variations as well. The most increases were noted in the Maldives, with a rise of 104.1% (95% UI: 73.3% to 146.4%), followed closely by Peru at 102% (95% UI: 50.5% to 164.4%) and Myanmar at 91.4% (95% UI: 58.5% to 131.1%). These statistics highlight the escalating burden of PCOS-related disabilities and further underscore the need for increased awareness and targeted healthcare interventions.

### Age and rei pattern

**Figure 3:**
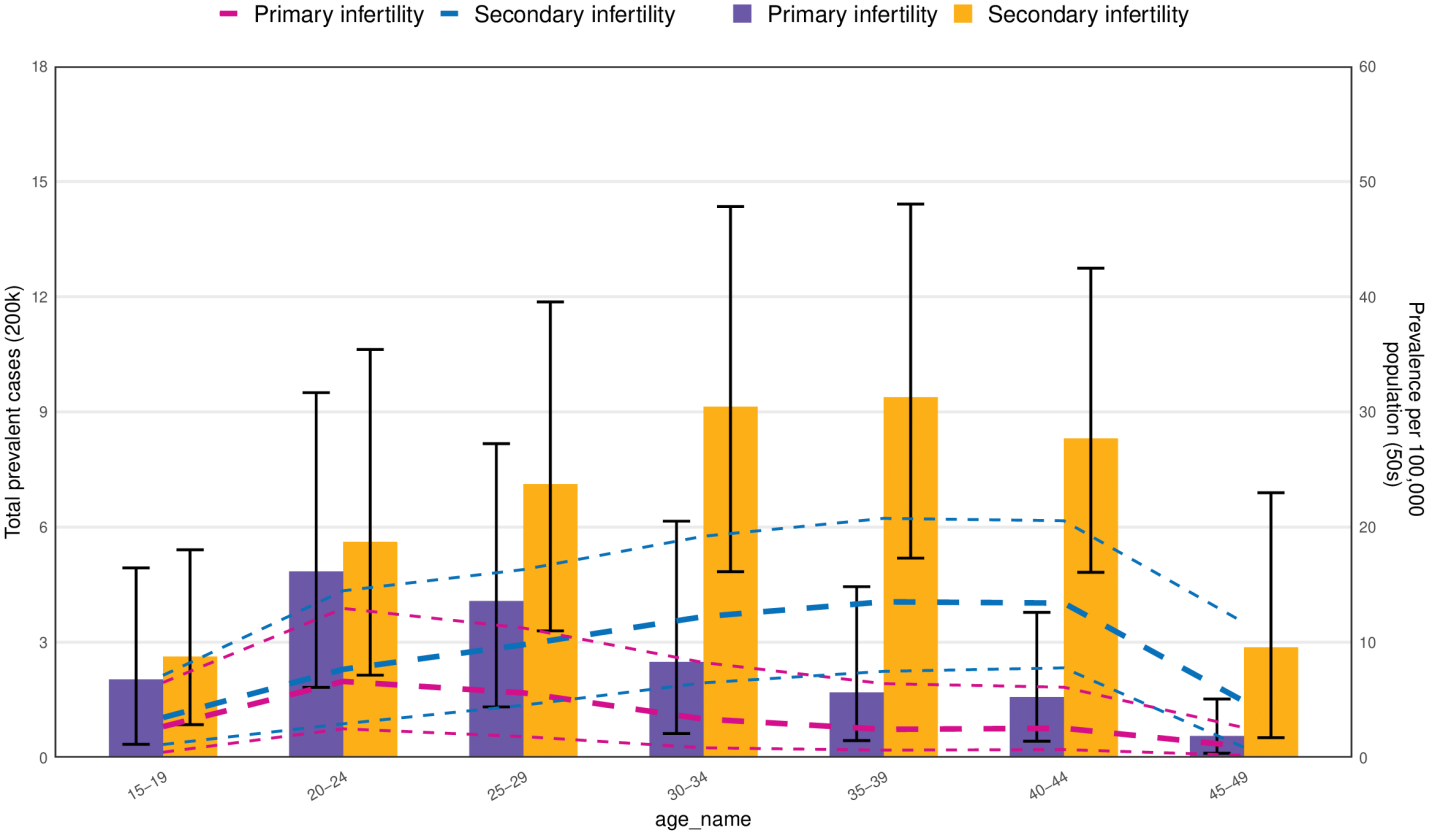
Number of prevalent cases globally and prevalence of PCOS-related infertility per 200,000 population, by age and subtype in 2021. Lines indicate prevalent cases with 95% uncertainty intervals for primary and secondary infertility.

In 2021, the highest global point prevalence of PRSI was observed in individuals aged 40–44 years. Conversely, the highest number of prevalent cases occurred in the 35–39-year-old age range, which remains elevated in primary infertility throughout all age categories (**Figure 3**). For primary infertility, both the global point prevalence and the number of prevalent cases peaked in the 20–24 years age group in 2021, followed by a decline in both metrics as age increased. Additionally, in 2021, the global YLD rates and the number of YLD of primary infertility increased up to age 25–29 years (**Supplementary Figure S1**),after which they diminished with older age groups. Whereas for PRSI, the YLD rates increased to the 40–44 age group. This rate was higher in primary infertility across all every age group. Also, the number of YLD peaked in the 35–39 age group and showed higher values for primary infertility up to the 20–24 age group (**Supplementary Figure S1**).

### The burden of PCOS-related infertility by SDI

**Figure 4:**
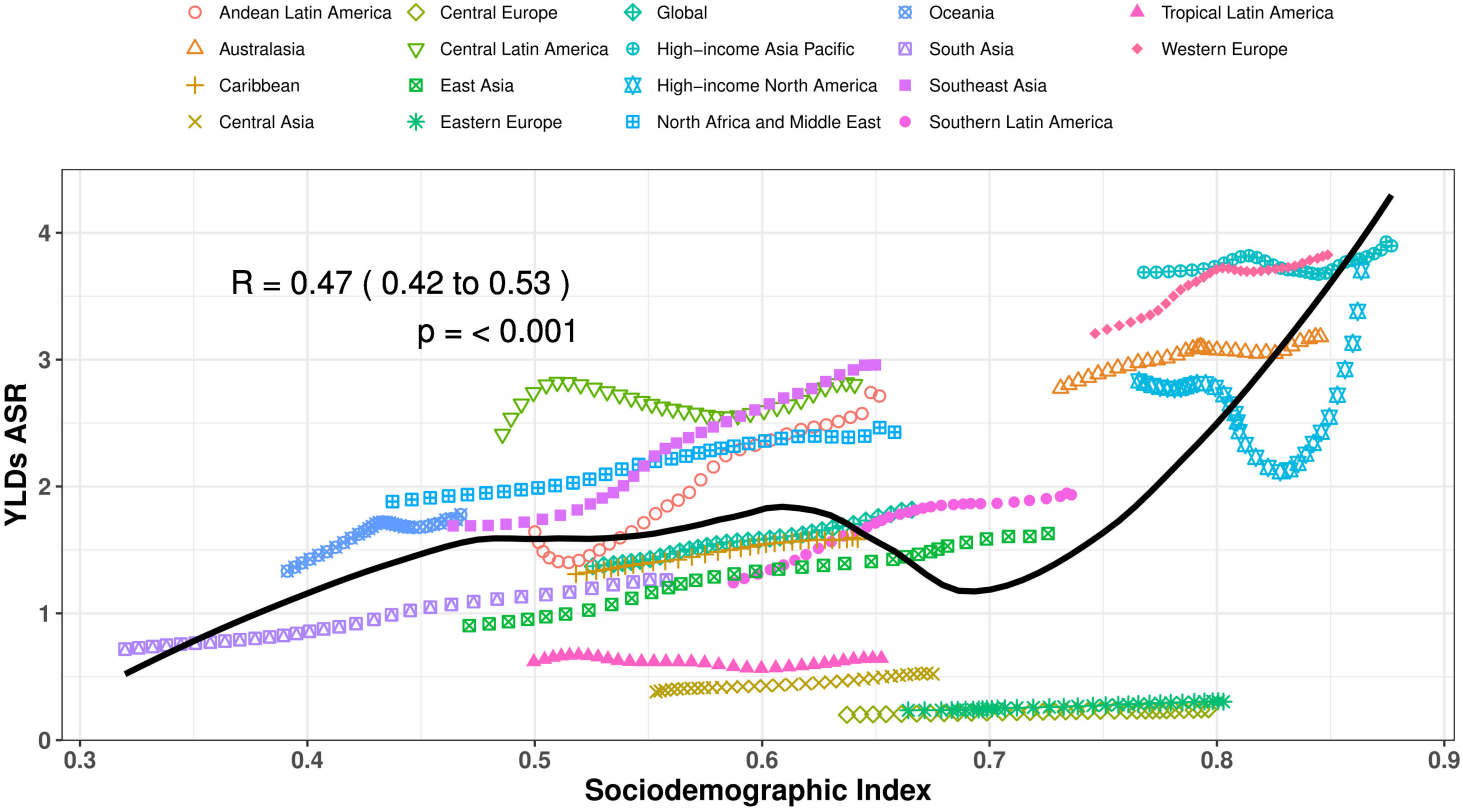
Age-standardized YLD rates of PCOS-related infertility for the 21 global burden of Disease regions by sociodemographic index, 1990–2021. Thirty-two points are plotted for each region and show the observed age-standardized YLD rates from 1990 to 2021 for that region. Expected values, based on sociodemographic index and burden estimates rates in all locations, are shown as a solid line. Regions above the solid line represent a higher-than-expected burden (eg, Southeast Asia), and regions below the line show a lower-than-expected burden (eg, Eastern Europe).

At the regional scale, over the period 1990–2021, we observed a steadily rising correlation between the SDI and the age-standardized YLD rates due to infertility. The age-standardized YLD rates improved exponentially with increases in SDI, up to about 0.6, and then declined briefly (**Figure 4**). During this period of decrease, regions like Southern Latin America, East Asia, and the Global average exhibited YLD rates that were higher than anticipated, based on their sociodemographic index. At approximately a sociodemographic index of 0.7, the age-standardized YLD rates increases significantly with the sociodemographic index, and the disease burden associated with PCOS-related infertility also rapidly increases (**Figure 4**).

During the rapid upward trend, Western Europe consistently had a higher-than-expected age-standardized YLD rates for PCOS-related infertility, from 1990 to 2021.Additionally, regions such as Central Latin America, North Africa, the Middle East, Southeast Asia, Western Europe, and Oceania recorded YLD rates above the expected threshold between 1990 and 2021. In contrast, Eastern Europe, Central Europe, Central Asia, Tropical Latin America, and the Caribbean showed YLD rates that fell below expectations during the measurement period. In more recent years, the Global average, along with Southern Latin America and East Asia, has displayed a level of disease burden that was above what was anticipated, whereas these regions had faced lower burdens at the earlier stages of the assessment period.

At the country level, a correlation was observed between the level of socioeconomic development and the age-standardized YLD rates of infertility linked to PCOS in 2021. Countries and territories, developed regions, such as New Zealand, Belgium, Israel, Australia, Iceland, and Japan had much higher-than-expected burdens. Less developed regions such as Angola, Comoros, Burundi, Lesotho, Guinea, and Nepal had significantly lower than expected burdens (**Supplementary Figure S2**).

### The BAPC model

**Figure 5:**
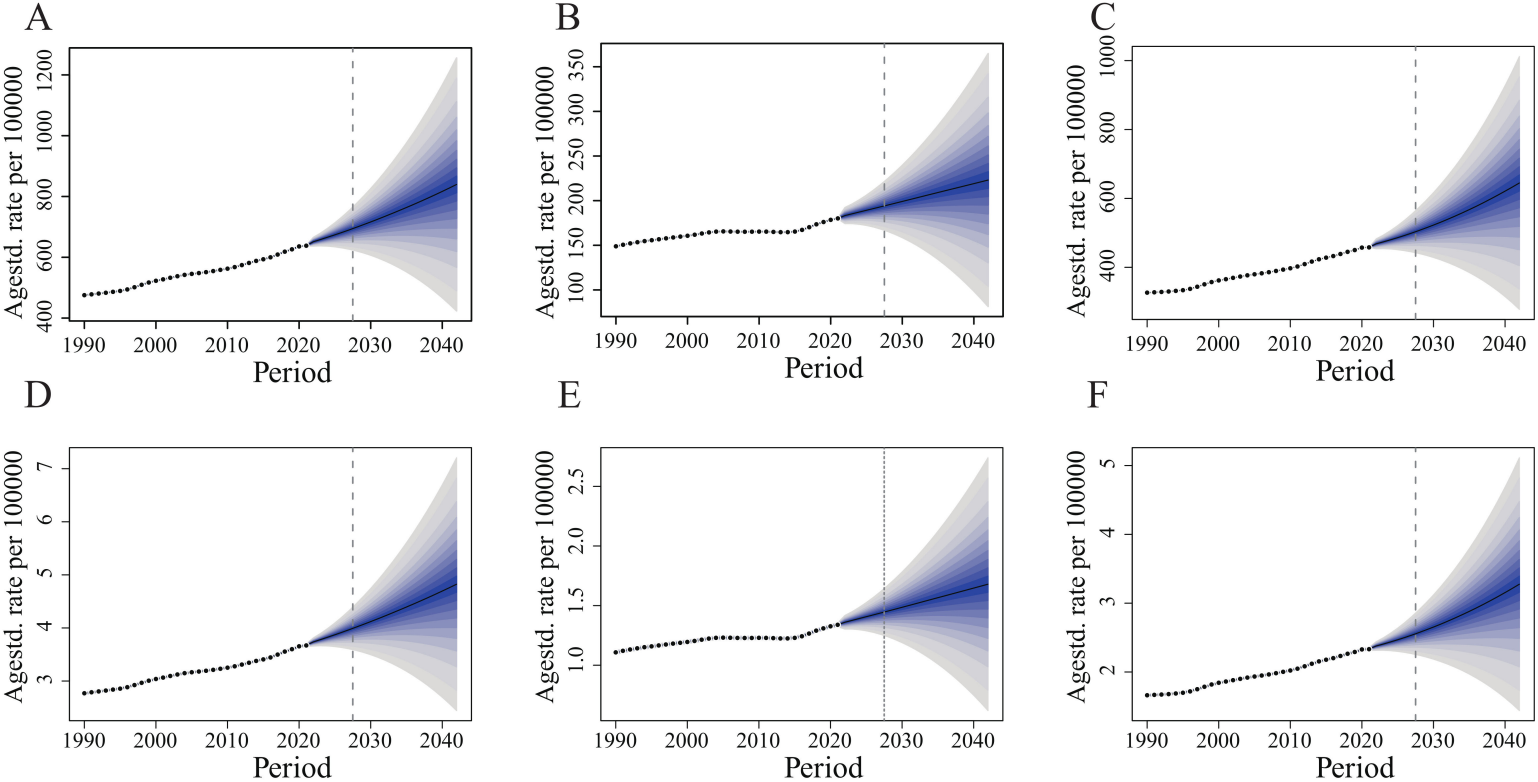
Observed and predicted trends of age-standardized prevalence and YLDs of infertility attributed to PCOS from 1990 to 2042 using the BAPC model. **(A)** Age-standardized prevalence of PCOS-related infertility. **(B)** Age-standardized prevalence of primary infertility. **(C)** Age-standardized prevalence of secondary infertility. **(D)** Age-standardized YLDs of PCOS-related infertility. **(E)** Age-standardized YLDs of primary infertility. **(F)** Age-standardized YLDs of secondary infertility.

We conducted a BAPC analysis for infertility attributable to PCOS and predicted the numbers of prevalence and YLD from 2022 to 2042 globally. The prediction model from BAPC indicated that from 2022 to 2042, a general rise in the age-standardized prevalence and YLD associated with various forms of infertility is anticipated. The most notable increase in age-standardized prevalence is anticipated in infertility, with projections indicating a rise from approximately 638 per 100,000 individuals to 840 per 100,000 individuals (**Figure 5A** **).** Similarly, secondary infertility is projected to experience a considerable rise in age-standardized prevalence, moving from 458 per 100,000 population to 645 per 100,000 population (**Figure 5C**). In contrast, primary infertility is expected to exhibit a stable trend in age-standardized prevalence, with a minor increase forecasted from about 180 cases per 100,000 in 2021 to 223 cases per 100,000 by 2042 (**Figure 5B**).

The age-standardized YLD rate for infertility related to PCOS is projected to stay fairly steady, showing a minor increase from 3.68 per 100,000 population in 2021 to roughly 4.82 per 100,000 by 2042 (**Figure 5D**). Likewise, secondary infertility is anticipated to show a relatively constant age-standardized prevalence, rising from 2.34 per 100,000 individuals to 3.28 per 100,000 individuals (**Figure 5F**). For primary infertility, the age-standardized prevalence is predicted to also remain stable, with a growth from about 1.34 per 100,000 in 2021 to 1.68 per 100,000 by 2042 (**Figure 5E**).

Based on the above indicators, secondary infertility consistently shows higher rates compared to primary infertility. Meanwhile, the gap between primary infertility and secondary infertility is extending over time, particularly when it comes to prevalence.

## Discussion

This research presents current insights on the prevalence and YLD associated with PCOS-related infertility, utilizing information from the GBD 2021 report. The results include age-standardized rates across 204 nations and territories, spanning from 1990 to 2021 (31). Our analysis indicates that the age-standardized point prevalence and YLD rates, along with the absolute counts, due to PCOS-related infertility have risen over the last 30 years. Secondly, the peak in age-standardized prevalence for secondary infertility is demonstrated by the 40–44 age cohort, whereas the highest absolute number of prevalent cases is held by the 35–39 age group. In contrast, the 20–24 age group is subjected to the heaviest burden associated with primary infertility. A similar phenomenon is observed in the YLD indicator. Thirdly, our research shows that the disease burden of PCOS-related infertility exhibits an increasing trend in the next 2 decades.

Globally, PCOS-related infertility accounted for 124.7 million prevalent cases and 71.6 thousand YLD in 2021. The age-standardized point prevalence and YLD rates, along with the absolute counts, due to PCOS-related infertility have increased over the last 30 years, which could be the result of the rapid increase in global obesity rate and the continuously expanded diagnostic criteria of PCOS (32–35). HJ Teede et al. discovered that each increase in body mass index(BMI) resulted in a 9.2% increase in the chance of diagnosing PCOS, which indicated that BMI emerged as the most significant correlate with PCOS status (36, 37). Furthermore, a study revealed that infertility-related stress and quality of life among women with PCOS are correlated with BMI, suggesting that reducing BMI could improve their quality of life and facilitate stress adaptation (38). Whatever, for women, one study showed the obesity rates increased by about 20% from the 2011/12 to 2017/20 cycle and the severe obesity rates increased by about 35% (39).

What’s more, the prevalence of PCOS is influenced by varying diagnostic criteria (40). Bozdag et al. reviewed a total of 55 studies on prevalence. According to the diagnostic criteria established by the National Institutes of Health, Rotterdam, and AE-PCOS Society, the prevalence rates of PCOS were found to be 6%, 10%, and 10%, respectively (41). It indicates that there may be some patients with PCOS who have not been diagnosed, resulting in a lower prevalence of PCOS-related infertility than the real situation. Therefore, comprehending the long-term impact of the disease is essential for emphasizing the significance of preventing infertility associated with PCOS from the outset. The peak prevalence and YLDs for primary infertility occur at ages 20 to 24. Notably, this is consistent with the diagnostic criteria for PCOS in adults because of the overlap in normal physiological changes during puberty, such as irregular menstrual cycles, acne, and polycystic ovary morphology observed on pelvic ultrasound (42). PCOS is not fully diagnosed during adolescence (43, 44). Due to the complexity of PCOS diagnosis among young people, the real burden of infertility is more complicated and severe than what the data shows. Furthermore, the treatment of PCOS is frequently temporary and constrained by the absence of knowledge about the etiology and underlying mechanisms of the condition (45, 46). Therefore, targeted and effective strategies are needed to prevent and treat infertility and PCOS in age groups with high PCOS burden. Meanwhile, it is necessary to carry out active treatment and long-term management for patients with PCOS-associated infertility.

Earlier research has revealed a rising incidence of infertility linked to PCOS in 2019; however, there is a lack of studies predicting future prevalence rates and data specifically regarding 2021 (15). Our study compiles 30 years of global PCOS-related infertility across age groups, disease data and shows trends, allowing multidimensional analysis of spatiotemporal exposure. The purpose of this study was to gain a clearer insight into the current burden of PCOS-related infertility.

The findings of this study hold significant implications for public health and clinical practice. Firstly, this study is the first to explore the global, regional, and national burden of infertility attributed to PCOS, utilizing data from the GBD 2021, which offers thorough and timely insights from diverse sources. The extensive integration of data not only addresses data deficiencies in specific regions but also furnishes global health policymakers with a cohesive reference framework. Secondly, we assessed the infertility burden linked to PCOS across various age groups and subtypes, which reflects the burden situation of different subtypes. This underscores the critical need for early detection and screening programs, as well as public awareness initiatives focused on the signs and symptoms of PCOS-related infertility. By identifying high-risk groups and regions through detailed analysis, it provides a robust scientific foundation for implementing targeted public health interventions. Thirdly, we forecasted the disease burden of PCOS-related infertility for the next two decades, highlighting the need for targeted prevention and control strategies. This forward-looking analysis equips policymakers with projections of future disease burden, facilitating early planning and optimal allocation of healthcare resources.

## Limitations

Several constraints create opportunities for further enhancement of this research. First, information regarding PCOS and related infertility in certain countries and regions necessitates additional refinement to yield a more precise and rational estimate of the disease’s impact. Currently, the disease burden and associated harm do not encompass the full age spectrum, resulting in the omission of crucial data. Considering that our research spans three decades, the criteria for diagnosing and confirming the disease have evolved during this interval. Moreover, there exists no standardized guideline for diagnosing PCOS currently, which may contribute to instances of overdiagnosis or underdiagnosis. Consequently, we needed to take into account several false-positive and false-negative results that emerged. This constraint might have resulted in an alternative perspective compared to other existing studies thus far. Secondly, this study does not provide a comprehensive understanding of the factors that affect infertility related to PCOS, including environmental factors, dietary practices, lifestyle decisions, or metabolic concerns. The inclusion of this supplementary data would enhance the understanding of the burden posed by PCOS.

## Conclusions

The research we conducted highlights the global impact of infertility related to PCOS and the need for effective management and prevention of this condition. Over the last 3 decades, the prevalence of PCOS has notably risen, particularly within the age brackets of 20-24, 35-39, and 40-44. Our analysis indicates a growing trend in the prevalence of PCOS, expected to continue over the next 2 decades. These results highlight the urgent need to improve strategies for managing and preventing PCOS in several nations. To address the increasing prevalence and YLD of PCOS-related infertility, we advocate a collaborative approach that includes input from various disciplines and the development of interconnected medical record systems to enhance PCOS management.

## Data Availability

Publicly available datasets were analyzed in this study. This data can be found here: The datasets analysed during the current study are available in the public databases: The GBD 2021 database (https://vizhub.healthdata.org/gbd-results/).

## Supplementary material

Supplementary Figure 1 Number of YLD cases globally and YLD rates of PCOS-related infertility per 500 population, by age and subtype in 2021. Lines indicate prevalent cases with 95% uncertainty intervals for primary and secondary infertility.

Supplementary Figure 2 Age-standardized YLD rates of PCOS-related infertility for the 204 countries by sociodemographic index, 1990–2021. 204 points are plotted for each countries and show the observed age-standardized YLD rates from 1990 to 2021 for that country. Expected values, based on sociodemographic index and burden estimates rates in all locations, are shown as a solid line. Countries above the solid line represent a higher-than-expected burden, and countries below the line show a lower-than-expected burden.
