# Supplementary figures and images for: The burden of polycystic ovary syndrome-related infertility in 204 countries and territories, 1990-2021: an analysis of the global burden of disease study 2021
Source: Front Endocrinol (Lausanne). 2025 Jun 6;16:1559246. doi: 10.3389/fendo.2025.1559246 (PMC12178856; doi:10.3389/fendo.2025.1559246)

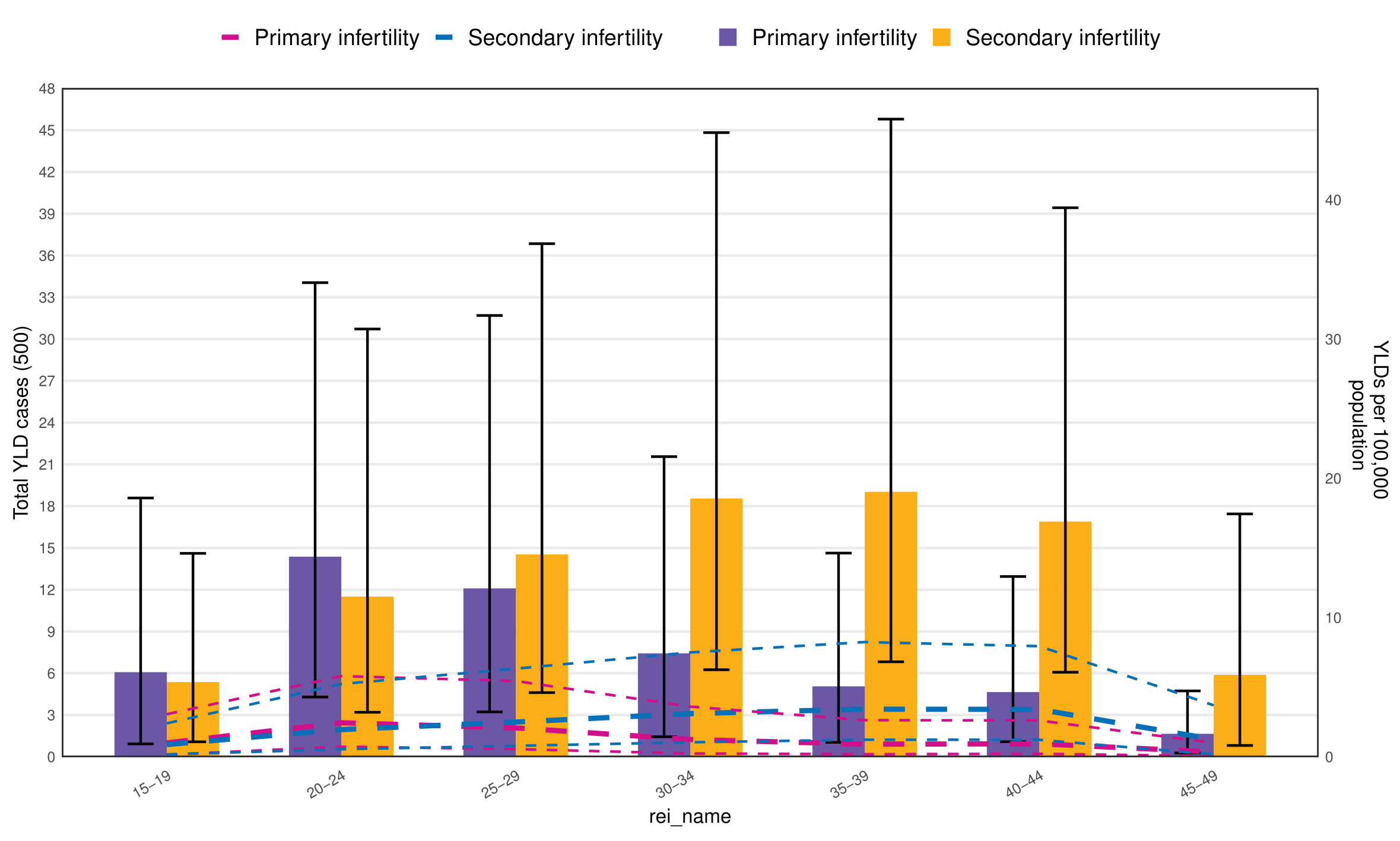

Supplement: Supplementary Figure 1 — Number of YLD cases globally and YLD rates of PCOS-related infertility per 500 population, by age and subtype in 2021. Lines indicate prevalent cases with 95% uncertainty intervals for primary and secondary infertility. [file Image1.tif]

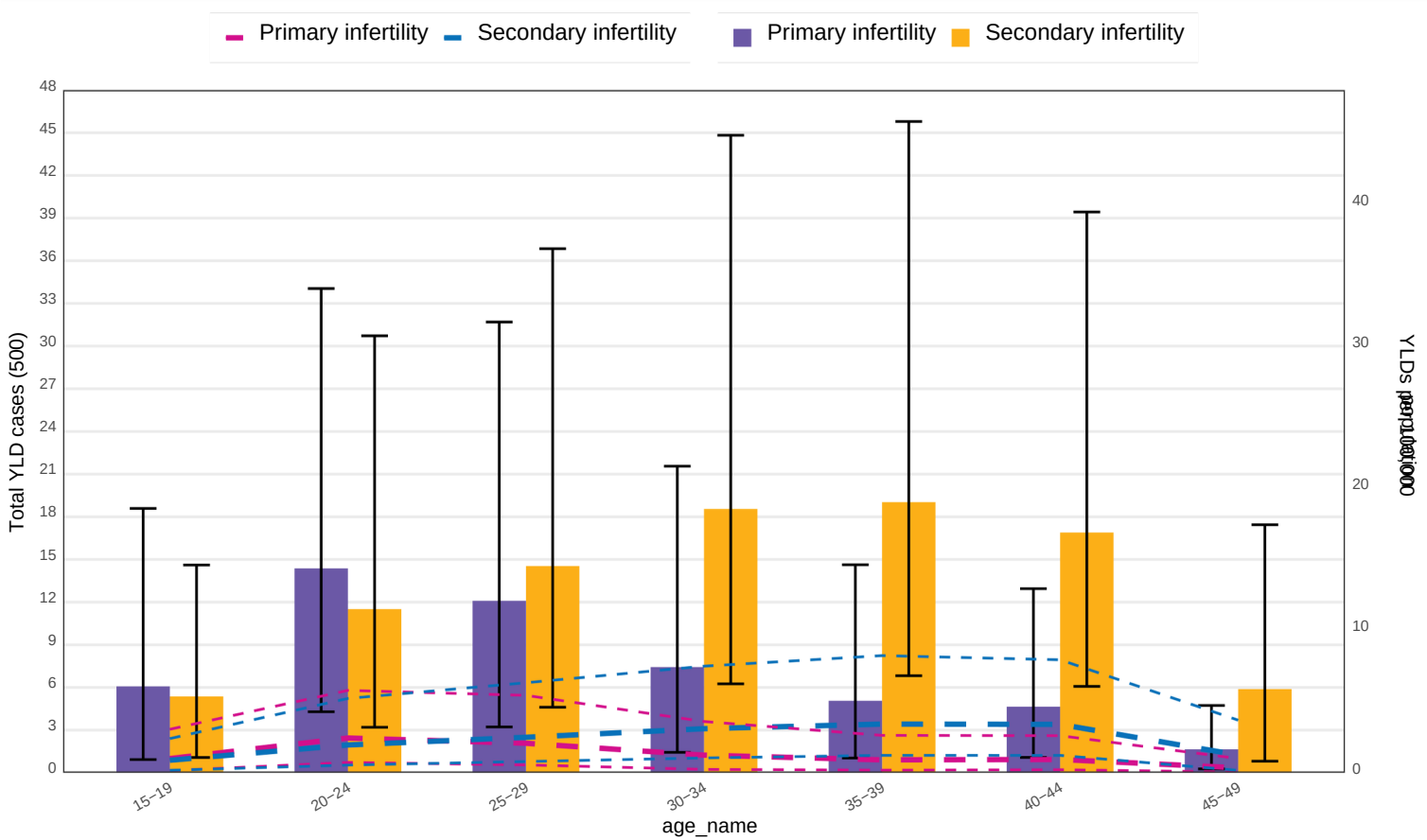

Supplement: Supplementary Figure 2 — Age-standardized YLD rates of PCOS-related infertility for the 204 countries by sociodemographic index, 1990–2021. 204 points are plotted for each countries and show the observed age-standardized YLD rates from 1990 to 2021 for that country. Expected values, based on sociodemographic index and burden estimates rates in all locations, are shown as a solid line. Countries above the solid line represent a higher-than-expected burden, and countries below the line show a lower-than-expected burden. [file DataSheet1.pdf]
