# Supplementary material for: The burden of polycystic ovary syndrome-related infertility in 204 countries and territories, 1990-2021: an analysis of the global burden of disease study 2021
Source: Front Endocrinol (Lausanne). 2025 Jun 6;16:1559246. doi: 10.3389/fendo.2025.1559246 (PMC12178856; doi:10.3389/fendo.2025.1559246)
Supplement: Supplementary file 3 [file DataSheet2.pdf]

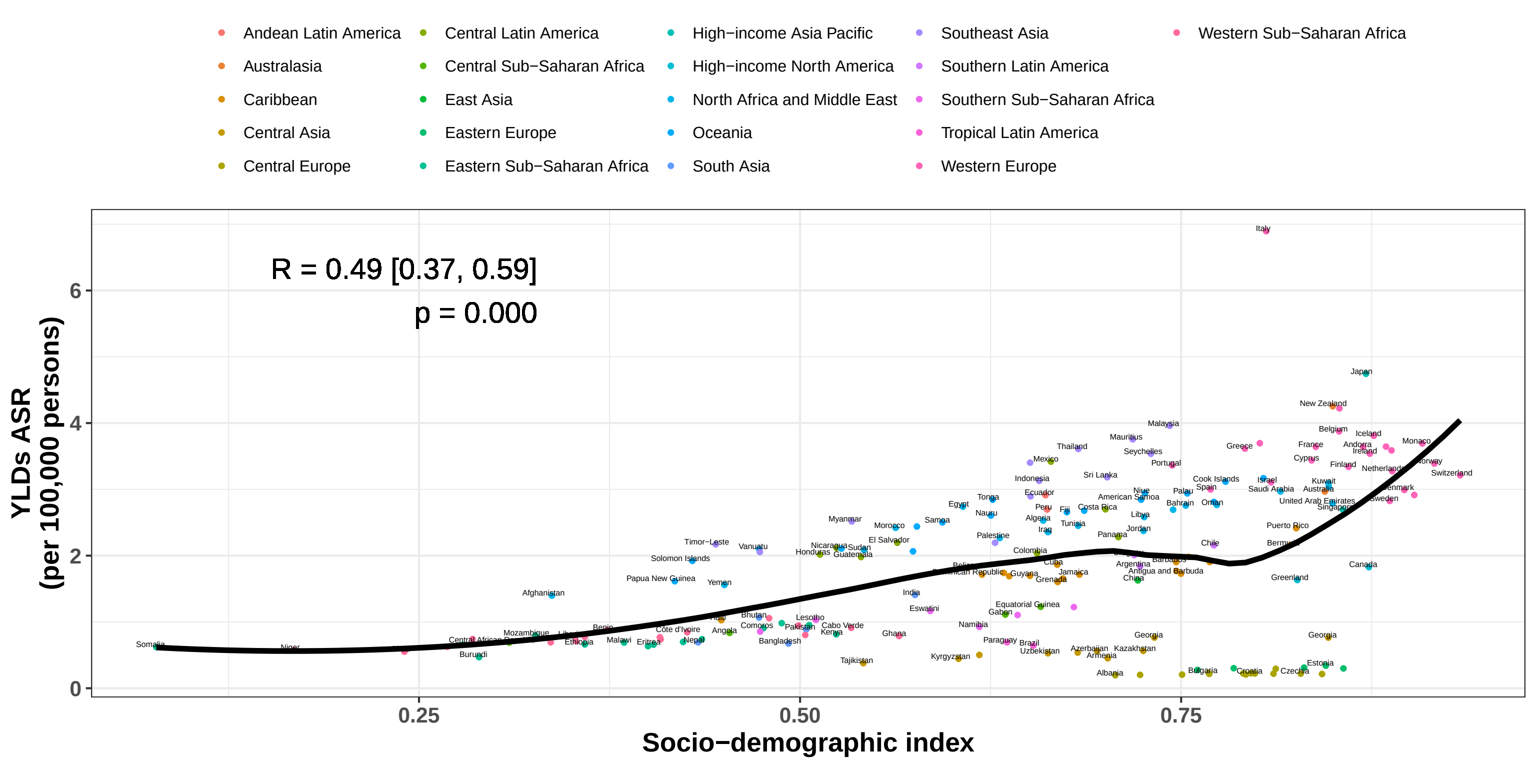

Andean Latin America
Australasia
Caribbean
Central Asia
Central Europe
Central Latin America
Central Sub−Saharan Africa
East Asia
Eastern Europe
Eastern Sub−Saharan Africa
High−income Asia Pacific
High−income North America
North Africa and Middle East
Oceania
South Asia
Southeast Asia
Southern Latin America
Southern Sub−Saharan Africa
Tropical Latin America
Western Europe
Western Sub−Saharan Africa
R = 0.49 [0.37, 0.59]
p = 0.000
YLDs ASR
(per 100,000 persons)
0
2
4
6
0.25
0.50
0.75
Socio−demographic index
Italy
Japan
New Zealand
Malaysia
Belgium
Iceland
Monaco
Mauritius
Thailand
Greece
France
Andorra
Ireland
Seychelles
Mexico
Portugal
Cyprus
Finland
Netherlands
Norway
Switzerland
Sri Lanka
Indonesia
Cook Islands
Israel
Kuwait
Spain
Saudi Arabia
Australia
Denmark
Ecuador
Niue
Palau
Tonga
American Samoa
Sweden
Egypt
Peru
Fiji
Costa Rica
Bahrain
Oman
United Arab Emirates
Singapore
Nauru
Libya
Myanmar
Algeria
Samoa
Morocco
Tunisia
Iraq
Jordan
Puerto Rico
Palestine
Panama
El Salvador
Chile
Bermuda
Timor−Leste
Vanuatu
Nicaragua
Sudan
Honduras
Guatemala
Colombia
Solomon Islands
Cuba
Argentina
Barbados
Canada
Antigua and Barbuda
Guyana
Jamaica
Grenada
China
Greenland
Papua New Guinea
Yemen
Afghanistan
India
Eswatini
Equatorial Guinea
Gabon
Bhutan
Lesotho
Namibia
Comoros
Cabo Verde
Haiti
Côte d'Ivoire
Angola
Pakistan
Kenya
Ghana
Georgia
Mozambique
Benin
Malawi
Eritrea
Nepal
Ethiopia
Bangladesh
Paraguay
Brazil
Uzbekistan
Azerbaijan
Kazakhstan
Armenia
Somalia
Niger
Burundi
Tajikistan
Kyrgyzstan
Albania
Bulgaria
Croatia
Czechia
Estonia
